# Low Voltage Operating 2D MoS_2_ Ferroelectric Memory Transistor with Hf_1-x_Zr_x_O_2_ Gate Structure

**DOI:** 10.1186/s11671-020-03384-z

**Published:** 2020-08-02

**Authors:** Siqing Zhang, Yan Liu, Jiuren Zhou, Meng Ma, Anyuan Gao, Binjie Zheng, Lingfei Li, Xin Su, Genquan Han, Jincheng Zhang, Yi Shi, Xiaomu Wang, Yue Hao

**Affiliations:** 1 Wide Bandgap Semiconductor Technology Disciplines State Key Laboratory, School of Microelectronics, Xidian University, Xi’an, 710071 China; 2 School of Electronic Science and Engineering, Nanjing University, Nanjing, 210023 Jiangsu China

**Keywords:** Nonvolatile memory, Ferroelectric, MoS_2_, 2D, Field-effect transistor, HZO

## Abstract

Ferroelectric field effect transistor (FeFET) emerges as an intriguing non-volatile memory technology due to its promising operating speed and endurance. However, flipping the polarization requires a high voltage compared with that of reading, impinging the power consumption of writing a cell. Here, we report a CMOS compatible FeFET cell with low operating voltage. We engineer the ferroelectric Hf_1-x_Zr_x_O_2_ (HZO) thin film to form negative capacitance (NC) gate dielectrics, which generates a counterclock hysteresis loop of polarization domain in the few-layered molybdenum disulfide (MoS_2_) FeFET. The unstabilized negative capacitor inherently supports subthermionic swing rate and thus enables switching the ferroelectric polarization with the hysteresis window much less than half of the operating voltage. The FeFET shows a high on/off current ratio of more than 10^7^ and a counterclockwise memory window (MW) of 0.1 V at a miminum program (P)/erase (E) voltage of 3 V. Robust endurance (10^3^ cycles) and retention (10^4^ s) properties are also demonstrated. Our results demonstrate that the HZO/MoS_2_ ferroelectric memory transistor can achieve new opportunities in size- and voltage-scalable non-volatile memory applications.

## Background

The system on chip (SoC) embedded memory market is currently in an era of tremendous growth, which requires the memory are capable of achieving faster operation, smaller cell size, and less power consumption [1–6]. Ferroelectric memory, one of the most promising candidates, has been reconsidered, due to the discovery of ferroelectric hafnium oxide in 2011 [7].

In the past decades, FeFET did not perform well in all these aspects includes low voltage requirements for memory operation, process step’s simplicity, and minimally complementary metal-oxide-semiconductor (CMOS) integration process and limited contamination concerns [8–11]. To address this, recently, tremendous investigation on 2D FeFET nonvolatile memory (NVM) has been performed based on various ferroelectric materials, including PbZrTiO_3_ (PZT), and [P(VDF-TrFE)] polymer [12–18], which is due to the promising properties of 2D material in “more than Moore era.” In the FeFET, the two stable spontaneous polarization states of a ferroelectric material incorporated into a transistor gate stack are utilized for data storage via the controllable threshold voltage enabled by applied shrunken P/E gate voltages. It is reported that the reproducible hysteresis behaviors, a high on/off ratio of 10^4^, good retention properties up to 10^4^ s, and stable switching operation have been achieved in PZT/MoS_2_ FeFET [19]. Noticeably, a maximum mobility of 625 cm^2^/V∙s, a large MW of 16 V for a ± 26 V gate—voltage range and a high on/off ratio of 8 × 10^5^ have also been demonstrated by an n-type [P(VDF-TrFE)] polymer/MoS_2_ FeFET [15]. However, there are so many fundamental issues, which could prevent its practical application, like, CMOS compatibility, scaling capability, and the interface states between Fe and 2D material. Ferroelectric hafnium oxide, a kind of novel ferroelectric material, has excellent CMOS compatibility and scaling capability, which could serve for the advanced FeFET NVM at sub-5 nm technology node in the next 5-10 years [20]. Accordingly, a batch of HfO_2_-based dielectric stacks have been incorporated into 2D FeFETs, which are targeted to achieve negative capacitance field-effect transistors (NCFET) with steep ON/OFF switching via sub-60 mV/decade slope and hysteresis-free characteristics [21–26], Although mass experiments based on NC dielectric stack with alternate 2D channel materials have drawn fantastic conclusions, they highlighted the surge requirements to distinguish between NCFETs and FeFETs. There is still a lack of systematical investigation regarding the physics and viability of the device technology on one-transistor ferroelectric memory based on MoS_2_ and ferroelectric HZO.

In this work, a FeFET with a few-layered HZO MoS_2_ transistor has been proposed. It is capable of scaling the P/E voltage via the NC effect induced by gate stack engineering under a shrunken P/E voltage. We experimentally demonstrated that a counterclockwise MW of 0.1 V with sub-60 mV/decade slope has been achieved in HZO MoS_2_ FeFET, which can be attributed to local carrier density modulation in the 2D channel by fast flipping of ferroelectric dipole. We attributed the decreased hysteresis of the HZO/MoS_2_ FeFET as drain voltage increasing to negative drain-induced barrier lowering (DIBL) effect. In addition, it was also systematically studied retention, endurance characteristics, and the dependence of the threshold voltage on the drain voltage of HZO MoS_2_ FeFET, opening a feasible pathway to design HZO MoS_2_ FeFET NVM and its practical applications.

## Methods

6 nm Hf_1-x_Zr_x_O_2_ film and 2 nm Al_2_O_3_ was deposited on p^+^ Si substrate using ALD at 300 °C, with [(CH_3_)_2_N]_4_Hf(TDMAHf), [(CH_3_)_2_N]_4_Zr(TDMAZr), and H_2_O vapor as the Hf precursor, Zr precursor, and oxidant precursor, respectively. Subsequently, the substrate underwent rapid thermal annealing (RTA) at 450 °C for 30 s in N_2_ ambient. After that, few-layer MoS_2_ flakes were mechanically exfoliated and transferred onto the substrate. The diameter of p^+^ Si substrate used to deposit HZO (6 nm)/AI_2_O_3_ (2 nm) is 6 inches. We employed electron beam lithography (EBL) to pattern contact pads in poly(methyl methacrylate) (PMMA) A5 resist. The spin parameters, baking parameters, and imaging parameters are 500 r/min (9 s) + 4000 r/min (40 s), 170 °C (5 min), MIBK:IPA = 1:3 (15 s), respectively. Then, the source/drain electrodes (Ti/Au, 5/65 nm thickness) were evaporated using an e-beam evaporation (EBE) system and etched by acetone solution. After lift-off, the device was annealed at 300 °C for 2 h to enhance the contact. We carried out the electrical characterization of our fabricated MoS_2_/HZO field-effect transistors using a probe station with a micromanipulator. The back gate voltage (*V*_GS_) was applied on the p type heavily doped Si substrate. A semiconductor characterization system (PDA) was used to measure the source-drain voltage (*V*_DS_), the back gate voltage (*V*_GS_), and the source−drain current (*I*_DS_).

## Results and Discussion

**Fig. 1:**
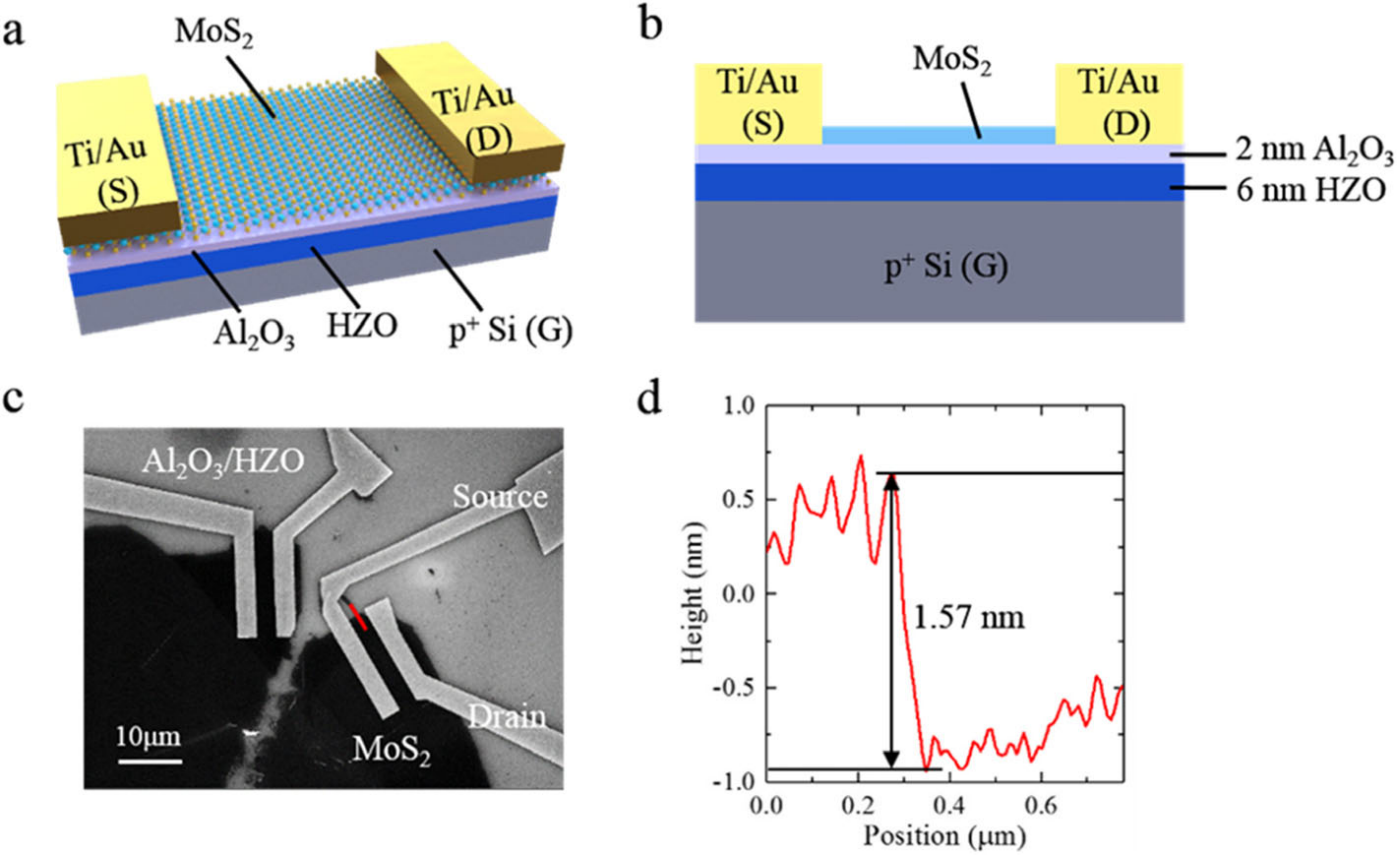
Device structure and basic properties of the MoS_2_/HZO FeFET. **a** Three-dimensional schematic representation of the MoS_2_/HZO FeFET. **b** Schematic cross section of the MoS_2_/HZO FeFET. **c** Top-view SEM image of the fabricated MoS_2_/HZO FeFET with Ti/Au source/drain electrodes, HZO ferroelectric gate insulators, and MoS_2_ channels. **d** Height profile using contact-mode AFM along the red line in **c**, validating the height of the MoS_2_ channel.

We prepared a few-layer MoS_2_ by mechanical exfoliation of bulk crystal and transferred the MoS_2_ nanoflake onto the 2 nm Al_2_O_3_/6 nm HZO/p^+^ Si substrate (see more details in the “Experimental” section). Figure 1a and b display a 3D schematic view and cross section of the HZO/MoS_2_ FeFET structure, respectively. A top-view scanning electron microscopy (SEM) image of the HZO/MoS_2_ FeFET is shown in Fig. 1c. The width and length of the MoS_2_ channel are 2 μm and 12 μm, respectively. As shown in Fig. 1d, the thickness of the MoS_2_ channel was confirmed using atomic force microscopy (AFM). The measured thickness of 1.57 nm indicates the presence of 4 layer of MoS_2_ [26].

As shown in Fig. S1c and d, the elemental and bond composition of HZO was examined by the X-ray photoelectron (XPS) measurements. Peaks are found to be 19.05 eV, 17.6 eV, 185.5 eV, and 183.2 eV, which correspond to the Hf 4f_5/2_, Hf 4f_7/2_, Zr 3d_3/2_, and Zr 3d_5/2_, respectively [27]. The atomic concentration along the depth profile in Fig. S1e further confirms the distribution of the Al_2_O_3_/HZO/p^+^ Si tri-layer structure. All the above confirm that the HZO film grown via our atomic layer deposition (ALD) system is highly crystalline.

**Fig. 2:**
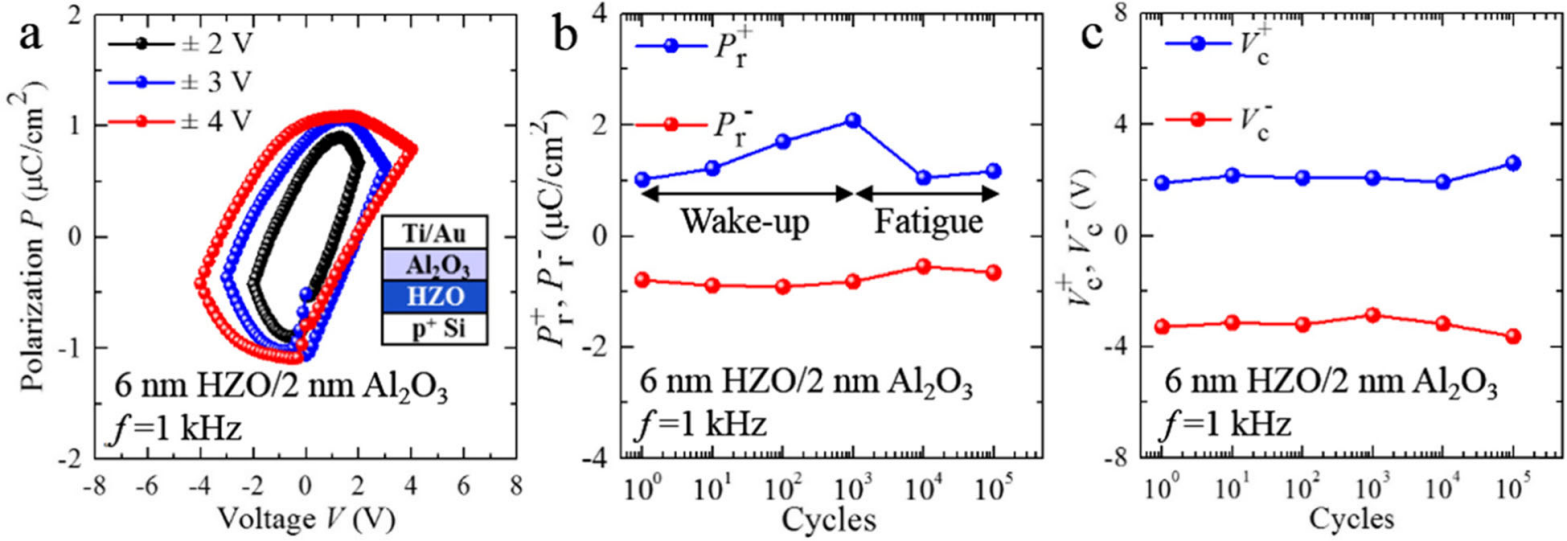
**a** P-V hysteresis loops for the HZO (6 nm)/Al_2_O_3_ (2 nm) capacitor with different voltage sweeping ranges. Dependence of (**b**) *P*_r_ and **c***V*_c_ on cycling for the HZO (6 nm)/Al_2_O_3_ (2 nm) capacitor with ± 4 V/1 kHz cycling

Before investigating the characterization of HZO/MoS_2_ FeFET, the ferroelectric behavior of the Au/2 nm Al_2_O_3_/6 nm HZO/p^+^ Si gate stack using polarization-voltage measurement is shown in Fig. 2a. Clearly, our fabricated 6 nm HZO/2 nm Al_2_O_3_ capacitors exhibit polarization-voltage hysteresis loops (measured at 1 kHz). Meanwhile, the remnant polarization *P*_r_ and the coercive voltage *V*_c_ increase with increasing the maximum sweeping voltage, implying the *P-V* hysteresis loops transform from minor loop to major loop. As the maximum sweeping voltage increases from 2 to 4 V, *P*_r_ reaches 0.66 μC/cm^2^, 0.86 μC/cm^2^, and 1.1 μC/cm^2^, respectively and *V*_c_ reaches 1.12 V, 1.9 V, and 2.04 V, respectively. Extracted *P*_r_ and *V*_c_ within 10^5^ enduring DC sweeping cycles are shown in Fig. 2b and c. Obviously, significant wake-up and fatigue effects within 10^5^ cycles are observed in the 6 nm HZO/2 nm Al_2_O_3_ capacitor. The wake-up and fatigue can be attributed to the diffusion and redistribution of the oxygen vacancies under the electric field. The fatigue effect is generally associated with charge trapping at the defect sites related to oxygen vacancies [28]. The hysteresis behaviors for the *PRphase* and butterfly-shaped loop for the *PRampl* using piezoresponse force microscopy (PFM) are displayed in Fig. S1b and c, indicating a polarization switching as a function of the sweep bias voltage. Considering different contact resistances between polarization-voltage measurement and piezo response-voltage measurement, the measured *V*_c_ in Fig. S1b and c is not so consistent with the values obtained in Fig. 2a.

Additionally, it is observed that there is an increase in MW accompanied with the raised sweeping voltage range of gate voltage (*V*_GS,range_). Usually, poly-crystal HZO film exists as multi-domain status [29], and the coercive field distribution of these domains satisfies Gaussian distribution. Thus, there must be an increased dependence on the raised *V*_GS,range_. The coercive filed *E*_*C*_ corresponds to the value of the external electric field which can reduce the remanent polarization to zero. Therefore, the *V*_GS_,_range_ used to switch the polarization in the HZO film becomes larger with higher related coercive voltage *V*_*C*_. This is the reason why polarization-voltage loops of HZO film are extended with a larger *V*_GS,range_, which has been demonstrated in Fig. 2a. In other words, the enhanced polarization intensity and ferroelectric switching occur with the raised *V*_GS,range_, leading to the aforementioned phenomena of the extended counterclockwise MW produced by the increased *V*_GS,range_. At *V*_GS,range_ = (−2, 2 V), the MW are almost vanished and nearly hysteresis-free characteristics emerge, which means the almost complete compensation between the effects of ferroelectric switching and charge trapping/de-trapping.

**Fig. 3:**
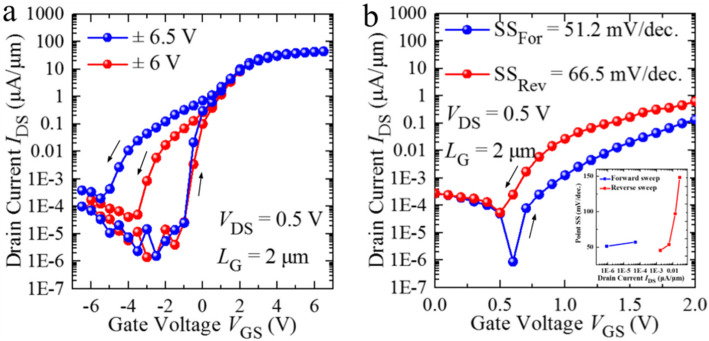
The direct current (DC) test of the HZO/MoS_2_ FeFET when drain voltage (*V*_DS_) is 0.5 V. **a** The comparison between transfer curves with 6 V and 6.5 V as maximum of the back gate voltage. **b** Enlarged view of transfer curves at 0 to −2 V interval of *V*_GS,range_ = (−3, 3 V). Point subthreshold slope (SS) as a function of drain current (*I*_DS_) of the HZO/MoS_2_ FeFET is (**b**) inset. The device exhibits SS_For_ = 51.2 mV/dec

In order to further investigate the effect of ferroelectric switching, the *V*_GS,range_ has been continuously increased to (−6, 6 V) and (−6.5, 6.5 V). The measured *I*_DS_-*V*_GS_ curves of the HZO MoS_2_ FeFET at *V*_GS,range_ = (−6, 6 V), and (−6.5, 6.5 V) are shown in Fig. 3a. Similarly, the counterclockwise memory window is increased with the extended *V*_GS,range_. At *V*_GS,range_ = (−6.5, 6.5 V), the counterclockwise MW is above 4 V and the on/off ratio also increases to 10^7^, which is due to the enhanced polarization switching under a larger external applied voltage. Generally, the mechanism underlying the hysteresis behaviors shown in the *I*_DS_-*V*_GS_ curves during the bi-direction sweeping of *V*_GS_ is threshold voltage shift, which can be modified by the predominant effects of polarization switching, that is NC effect [30–32], resulting in counterclockwise hysteresis. A further study of improved subthreshold characteristics was carried out in the other device under a shrunken *V*_GS,range_. The measured *I*_DS_-*V*_GS_ and extracted point SS—*I*_DS_ curves of the other device at *V*_GS,range_ = (−3, 3 V) are plotted in Fig. 3b. It is demonstrated that at *V*_GS,range_ = (−3, 3 V), HZO/MoS_2_ FeFET exhibits SS_For_ = 51.2 mV/decade and SS_Rev_ = 66.5 mV/decade, respectively. That is to say, the SS of sub-60 mV/decade and a MW of 0.48 V can be simultaneously achieved in HZO/MoS_2_ FeFET at room temperature, which will be a hint to distinguish between NCFET and FeFET.

As it is known, in NCFET, the SS can be smaller than 60 mV/decade at room temperature due to the incorporation of the negative gate dielectric capacitance (*C*_ins_), which can be obtained via the negative slope segment of d*P*/d*E* < 0 induced by ferroelectric film, contributing to the gate stack factor (m) < 1. The mechanism underlying the NC effect [33] is the depolarization field generated by ferroelectric film [34–38]. It is experimentally reported that due to the incomplete screening at the interface of ferroelectric film [39], the residual polarization charge could produce an internal electrical field across ferroelectric film, which has the opposite direction with the externally applied voltage, leading to the re-distribution of the voltage across the gate stack and the amplified channel surface potential, named as “voltage amplification effect” [40–42]. The voltage amplification usually can be divided into two parts, the accelerated variation of channel surface potential and the subsequent boosted value, providing the steep ON/OFF switching and improved *I*_ON_/*I*_OFF_, respectively. However, for FeFET, there is another story. According to the concept of capacitance matching between ferroelectric capacitance (*C*_FE_) and metal-oxide-semiconductor capacitance (*C*_MOS_) [43–45], when |*C*_FE_| > *C*_MOS_, the theoretical total capacitance (*C*_total_) is positive and the system is stable, resulting in the same polarization behaviors during the bi-direction sweeping of *V*_GS_ and the stable hysteresis-free NCFET. However, good matching resulting in improved SS and transconductance is very tricky to achieve, since both *C*_MOS_ and *C*_FE_ are very non-linear, bias dependent capacitors. Additionally, |*C*_FE_| > *C*_MOS_ needs to be ensured for all the operating voltage range to avoid hysteresis. Instead, once |*C*_FE_| < *C*_MOS_, the theoretical *C*_total_ is negative and the system is unstable, a separated polarization behavior must occur during the bi-switching of *V*_GS_ to keep the *C*_total_ positive, which could produce the counterclockwise hysteresis in FeFET for NVM application. Here, it is mentioned that the hysteretic behaviors is the subsequent effect of separated polarization switching, which means that the width of hysteresis window can be easily modified based on the concept of capacitance matching, such as, which can be manipulated by the variation of *V*_DS_. With an appropriate capacitance matching, even with a much shrunken *V*_GS,range_ = (−3, 3 V), HZO/MoS_2_ FeFET still exhibits an obvious hysteresis window, and the steep switching of SS_For_ = 51.2 mV/dec at the same time, which further suggests the existence of the NC effect (ferroelectric polarization effect) in the subthreshold region as well. Although NCFET and FeFET are different, FeFET can also be adopted as logic devices with a comparable smaller MW, maintaining a deep sub-60 mV/dec SS, and a higher *I*_ON_/*I*_OFF_ ratio as well due to NC effect.

The impact of *V*_DS_ on the width of MW has been carefully investigated. The *I*_DS_-*V*_GS_ curves on logarithmic scales under different *V*_DS_ are characterized in Fig. S3. It is exhibited that, at a fixed *V*_GS,range_ = (−2, 2 V), the values of *V*_GS_ extracted at *I*_DS_ = 70 nA for the bi-directional sweeping of *V*_GS_ all shift to the negative direction. Meanwhile, it is also demonstrated that the variation in forward sweeping of *V*_GS_ is much more obvious over that of reverse sweeping, indicating the significant phenomena of negative DIBL. It should be noted that the negative DIBL effect always occurs with a NC effect [46, 47].

**Fig. 4:**
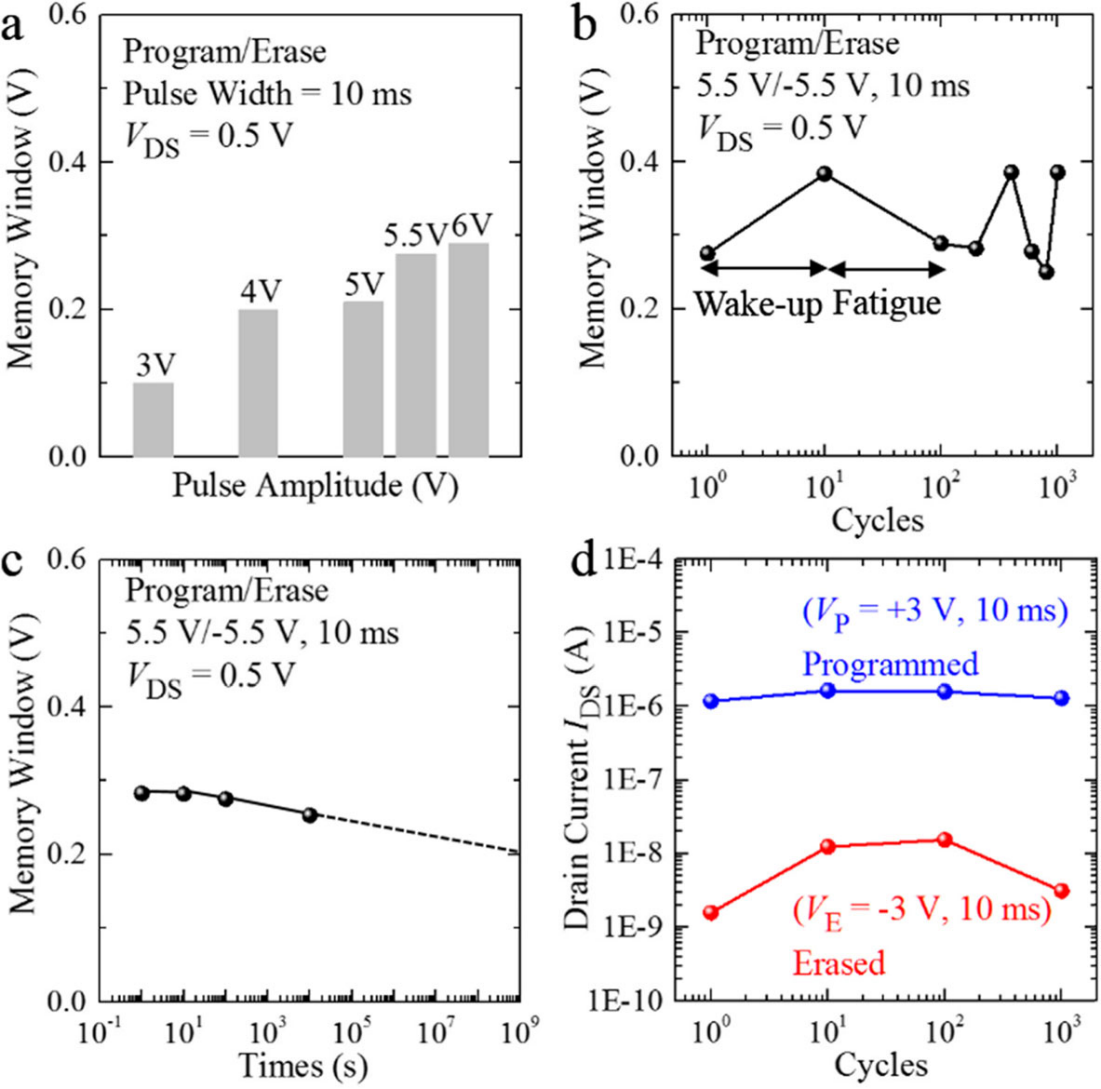
Memory performances of the HZO/MoS_2_ FeFET under P/E pulses. **a** Extracted MWs (MWs) under P/E pulses with ± 3 V, ± 4 V, ± 5 V, ± 5.5 V, and ± 6 V heights. **b** Endurance measurements under P/E pulse conditions. **c** Retention characteristic of the HZO/MoS_2_ FeFET. **d** Endurance of the HZO/MoS_2_ FeFET for 10^3^ cycles under the P/E pulses with ± 3 V heights

After the above direct current (DC) test of the HZO/MoS_2_ FeFET, we further carried out the measured MWs for different P/E *V*_GS_ pulses with 10 ms width in Fig. 4a. MW is defined as the maximum change Δ*V*_TH_ after P/E *V*_GS_ pulses. During the pulsed *V*_GS_ application, the other terminals were fixed to *V*_S_ = *V*_D_ = 0 V. For the read (R) operation, *V*_GS_ was ranged from −1 V to 1 V with *V*_D_ = 0.5 V and *V*_S_ = 0 V. As shown in Fig. 4a, the extracted MWs become larger as P/E *V*_GS_ pulses increase. When the imposed P/E *V*_GS_ pulse is ± 3 V, the extracted MW is 0.1 V. When the imposed P/E *V*_GS_ pulse is ± 5.5 V, the extracted MW is 0.275 V. Compared with the counterclockwise MWs of 4 V and 0.48 V in Fig. 3a and b, the extracted MWs after P/E *V*_GS_ pulse is greatly reduced. This is possibly due to a higher density of trapping states induced by high humidity in the air [48]. Thus, the charge trapping/de-trapping mechanism is enhanced and the counterclockwise hysteresis loop is decreased eventually. Furthermore, we studied the cycling endurance and data retention of the HZO/MoS_2_ FeFET under P/E pulses with ± 5.5 V height in Fig. 4b. The program *V*_GS_ pulse was 10 ms wide with *V*_S_ = *V*_D_ = 0 V. Figure 4b illustrates the measured MWs as a function of endurance cycles. The endurance cycle is formed by back-gate voltage periodic P/R/E/R pulses. Voltages applied to the back gate of the height of P, E, R were + 5.5 V, −5.5 V and 0 V, respectively. And the pulse width of P and E was 10 ms. Clearly, an MW of 0.3 V can be maintained without significant degradation after 10^3^ P/E cycles. As the number of endurance cycle increased, the MW increases to 0.38 V after 10 cycles and then decreases back to 0.28 V after 600 cycles. The first broaden MW is called wake-up effect and the later shrunken MW is called fatigue effect. The wake-up effect corresponds to domain-wall de-pinning, leading to an increase of switchable polarization domains of the HZO film [49]. The fatigue effect corresponds to newly injected charges that pin the domain walls after great numbers of P/E cycles [50]. The data retention at room temperature is shown in Fig. 4c. Here, the MW degradation is negligible after 10^4^ s. Therefore, a MW about 0.3 V can be expected to be sustainable over 10 years by the dotted extrapolation lines. As presented in Fig. 4d, the device is stable after 10^3^ cycles under the P/E pulses with ± 3 V heights. The stability of the HZO/MoS_2_ FeFET shows a great perspective of applications in nonvolatile memory technology.

A comparison of figure-of-merit with FeFET-based devices combining MoS_2_ and ferroelectric gate dielectrics is provided in Table 1. Here, the device structure, remnant polarization, coercive electric field, hysteresis loop direction, MW, working voltage, endurance cycles, and retention time are listed. It is obvious that the device we fabricated exhibits the thinnest ferroelectric layer of 6 nm HZO and the lowest working voltage compared with other works [12–18], which is important for the future 2 nm or 3 nm process node of the back end of line (BEOL) memory. By scaling the thickness of the ferroelectric layer, a MW of about 0.1 V was achieved under a low working voltage of ± 3 V. Such a low working voltage can be attributed to the intrinsic characteristics of HZO layer compared with their counterparts, such as P(VDF-TrFE) or HfO_2_, which has much higher thickness. Furthermore, our device possesses lower remnant polarization *P*_r_ of 1.1 μC/cm^2^ compared with other reported FeFETs. The fast decay of retention loss in a FeFET is due to the existence of depolarization field *E*_dep_, which comes from the incomplete charge compensation due to the existence of the Al_2_O_3_ layer. Here, *E*_dep_ is directly proportional to the remanent polarization *P*_r_ [51]. Thus, the high *E*_c_ and low *P*_r_ make the ratio *E*_dep_/*E*_c_ in MoS_2_/HZO FeFET much small, leading to a much small retention loss associated with the depolarization field effect. Although the retention performances of MoS_2_ FeFETs based on HZO and P(VDF-TrFE) are both around 10^4^ s, the P(VDF-TrFE) film needs to be 150 nm [17].

**Table 1:** Comparison among the figure of merits of ferroelectric FETs based on MoS_2_.

MoS_2_ (thickness)	Ferroelectric layer (thickness)	Control gate (position)	*P*_r_ (μC/cm^2^)	*E*_c_ (MV/cm)	Hysteresis	MW [V]	*Working voltage* [V]	Endurance [Cycles]	Retention [s]	Ref.
few layers	HZO (6 nm)	P^+^ Si (back)	1.1	1.62	Counter-clockwise	0.3	± 5.5	10^3^	10^4^	This work
3 L	PZT (100 nm)	SrRuO_3_ (back)	65	2	Counter-clockwise	20	(−10, 30)	—	—	[12]
4 L	PZT (260 nm)	Pt (back)	56.03	8	Clockwise	2.5	± 4			[13]
3 L	P(VDF-TrFE) (*≈* 300 nm)	Al (top)	7	0.75	Counter-clockwise	25	± 40	—	—	[14]
5 L	P(VDF-TrFE) (220 nm)	Au (top)	6.5	0.55	Counter-clockwise	16	± 26	—	600	[15]
1 L	P(VDF-TrFE) (200 nm)	Al (top)	10	0.5	Counter-clockwise	15	± 20	—	10^3^	[16]
Several layers	P(VDF-TrFE) (150 nm)	Pt/Si (back)	8	0.6	Counter-clockwise	16	± 26	10^3^	3 × 10^4^	[17]
1 L	Al doped HfO_2_ (16 nm)	P^+^ Si (back)	3	1.5	Counter-clockwise	0.125	± 10	2 × 10^4^	—	[18]
3 L	P(VDF-TrFE) (100 nm)	P^+^ Si (back)	40	0.3	Clockwise	4.5	± 6	500	10^4^	[19]

## Conclusions

In conclusion, we investigated few-layered, MoS_2_-based ferroelectric memory transistor devices using an HZO back gate dielectric. Our fabricated devices exhibit counterclockwise hysteresis induced by ferroelectric polarization. In addition, our HZO/MoS_2_ ferroelectric memory transistor displayed excellent device performances: a high on/off current ratio of more than 10^7^ and a counterclockwise MW of 0.1 V at a P/E voltage of 3 V, which has the endurance (10^3^ cycles) and retention (10^4^ s) performance. We thus believe that the results of our MoS_2_-based nonvolatile ferroelectric memory transistors exhibit promising perspectives for the future of 2D low-power non-volatile memory applications.

## Supplementary information

**Additional file 1: Supplementary Information.** Characterization of ferroelectric HZO substrate and more transfer curves of the HZO/MoS_2_ FeFET(PDF). **Fig. S1 a** Optical image of the MoS_2_/HZO FeFET. **b***PRphase* and **c***PRampl* of the HZO capacitor. XPS analysis of the 2 nm Al_2_O_3_/6 nm HZO/p^+^ Si shows pristine **d** Hf and **e** Zr peaks. **f** XPS depth profile of the Al_2_O_3_/HZO/p^+^ Si tri-layer structure. A top-view optic image of the HZO/MoS_2_ FeFET is shown in Fig. S1a. As shown in Fig. S1b and c, *PRphase* and *PRampl* of the HZO capacitor suggest ferroelectric behavior after 450 °C rapid thermal annealing (RTA) measured at 1kHz. As shown in Fig. S1d and e, the elemental and bond composition of HZO were examined by the X-ray photoelectron (XPS) measurements. Peaks are found to be 19.05 eV, 17.6 eV, 185.5 eV, and 183.2 eV, which correspond to the Hf 4f _5/2_, Hf 4f _7/2_, Zr 3d _3/2_ and Zr 3d _5/2_, respectively [27]. The atomic concentration along the depth profile in Fig. S1f further confirms the distribution of the Al_2_O_3_/HZO/p^+^ Si tri-layer structure. All the above confirm that the HZO film grown via our ALD system is highly crystalline. Fig. S2. Transfer curves of the HZO/MoS_2_ FeFET at increasing gate voltage (*V*_GS_) ranges with the linear y-axis. For a start, the transfer curves of the HZO/MoS_2_ FeFET under different back gate voltage sweep ranges (*V*_GS,range_) and different drain voltages (*V*_DS_) have been characterized in Fig. S2. It is demonstrated that, the counterclockwise hysteresis windows have been obtained at various gate voltage range (*V*_GS,range_) from (-5, 5V) to (-2, 2V). Simply, the mechanism underlying the hysteretic behaviors shown in the transfer curves during the bi-direction sweeping of *V*_GS_ is threshold voltage shift, which can be modified by the effects of trapping/de-trapping [52] and polarization switching [53]. If the applied voltage is not high enough to switch the polarization in HZO film, charge trapping/de-trapping mechanism dominates and will cause clockwise hysteresis. The energy band at the interface between the MoS_2_ channel and ferroelectric back gate tends to bend downward after the positive back gate voltage. The more traps located below the Fermi-level; the more electrons are captured close to the interface. This will increase the threshold voltage after the positive gate pulse. The energy band at the interface between the MoS_2_ channel and ferroelectric back gate tends to bend upward after the negative back gate voltage. The more traps locate above the Fermi-level; the more electrons are released close to the interface. This will decrease the threshold voltage after the negative gate pulse [52]. If the applied voltage exceeds the coercive voltage in the HZO film, ferroelectric polarization mechanism dominates and will cause anti-clockwise hysteresis window [54–57]. Thus, it is easily concluded that the electrical performance of the device shown in Fig. S2 is dominated by ferroelectric switching. When the back-gate sweeps are in small ranges of 2V in Fig. S2a, we observed the nearly hysteresis-free switching. The hysteresis loops in Fig. S2b are counterclockwise for the back-gate sweep range of 6 V (from -3 V to 3 V). The minimum voltage under the drain is *V*_GS_ – *V*_DS_ = 2 V at *V*_DS_ = 1 V, which should be larger than the coercive voltage *V*_c_ to switch the ferroelectric at the drain side. The estimated coercive voltage is consistent with *V*_c_ of 1.9 V when the maximum sweeping voltage is 3 V in Fig. 2a. When the applied voltage in HZO film exceeds +*V*_c_, the ferroelectric polarization points into the MoS_2_ channel. Therefore, the electron charges in the MoS_2_ channel accumulate and the threshold voltage decreases. When the applied voltage in HZO film exceeds –*V*_c_, the ferroelectric polarization points away from the MoS_2_ channel. Therefore, the electron charges in the MoS_2_ channel deplete and the threshold voltage increases. Nonetheless, we observed that the wider back-gate voltage range leads to larger counterclockwise hysteresis loops in Fig. S2c and d. Due to the increment of *V*_c_ in Fig. 2a with increasing applied voltage, the ferroelectric polarization switching in the HZO film can be enhanced with a larger shift in threshold voltage. Fig. S3 Transfer curves of the HZO/MoS_2_ FeFET on logarithmic scales with **a***V*_DS_ = 0.05 V, **b***V*_DS_ = 0.2 V, **c***V*_DS_ = 0.4 V. **d** Extracted back gate voltage *V*_GS_ when drain current (*I*_DS_) equals to 70 nA with different *V*_DS_. Notably, besides the impact of *V*_GS,range_, it is found that *V*_DS_ can definitely adjust the memory window as well, which requires a further investigation. The *I*_DS_-*V*_GS_ curves on logarithmic scales under different *V*_DS_ are characterized in Fig. S3. It is exhibited that, at a fixed *V*_GS,range_ = (-2, 2 V), the values of *V*_GS_ extracted at *I*_DS_ = 70 nA for the bi-directional sweeping of *V*_GS_ all shift towards the negative direction and the variation in forward sweeping of *V*_GS_ is much more obvious over that of reverse sweeping, indicating the significant phenomena of negative drain induced barrier lowering (DIBL) [46, 58–61]. Generally, DIBL is a conventional short channel effect. With a short enough channel length, the increased *V*_DS_ can easily pull down the barrier between source/drain and enable a negative shift of threshold voltage, which is the so called effect of DIBL. However, for a ferroelectric FeFET, an increased *V*_DS_ is capable of producing a reduction of channel surface potential via the coupling between gate and drain induced by the parasitic capacitance between gate and drain (CGD), which means a positive shift of threshold voltage and can be called as negative DIBL.

## Data Availability

The authors declare that the materials, data, and associated protocols are available to the readers, and all the data used for the analysis are included in this article.

## References

[1] Brewer JE, Gill M (2007) Nonvolatile memory technologies with emphasis on flash: a comprehensive guide to understanding and using NVM devices. Wiley-IEEE Press

[2] Maayan E, Dvir R, Shor J, Polansky Y, Sofer Y, Bloom I, Avni D, Eitan B, Cohen Z, Meyassed M (2002) A 512 Mb NROM flash data storage memory with 8 MB/s data rate. Solid-State Circuits Conference IEEE

[3] Jang JJJ, Kim HS, Cho W, Cho H, Kim J, Shim SI, Jang Y, Jeong JH, Son BK, Kim DW (2009) Vertical cell array using TCAT (terabit cell array transistor) technology for ultra high density NAND flash memory. Symposium on VLSI Technology, 192-193

[4] Kawahara T, Ito K, Takemura R, Ohno H (2012). Spin-transfer torque RAM technology: review and prospect. Microelectron Reliab.

[5] Wong HSP, Raoux S, Kim S, Liang J, Reifenberg JP, Rajendran B, Asheghi M, Goodson KE (2010). Phase change memory. Proc IEEE.

[6] Jeong DS, Thomas R, Katiyar RS, Scott JF, Kohlstedt H, Petraru A, Hwang CS (2012). Emerging memories: resistive switching mechanisms and current status. Rep Prog Phys.

[7] Böscke TS, Müller J, Bräuhaus D, Schröder U, Böttger U (2011). Ferroelectricity in hafnium oxide thin films. Appl Phys Lett.

[8] Hong YK, Jung DJ, Kang SK, Kim HS, Jung JY, Koh HK, Park JH, Choi DY, Kim SE, Ann WS (2007) 130 nm-technology, 0.25 μm2, 1T1C FRAM cell for SoC (system-on-a-chip)-friendly applications. VLSI Technology, Symposium on IEEE

[9] Yamaoka K, Iwanari S, Marakuki Y, Hirano H, Gohou Y (2004) A 0.9 V 1T1C SBT-based embedded non-volatile FeRAM with a reference voltage scheme and multi-layer shielded bit-line structure. International Solid-state Circuits Conference IEEE

[10] Kohlstedt H, Mustafa Y, Gerber A, Petraru A, Fitsilis M, Meyer R, Böttger U, Waser R (2005). Current status and challenges of ferroelectric memory devices. Microelectron Eng.

[11] Mikolajick T, Dehm C, Hartner W, Kasko I, Kastner MJ, Nagel N, Moert M, Mazure C (2001). FeRAM technology for high density applications. Microelectron Reliab.

[12] Lu Z, Serrao C, Khan AI, Clarkson JD, Wong JC, Ramesh R, Salahuddin S (2018). Electrically induced, non-volatile, metal insulator transition in a ferroelectric-controlled MoS_2_ transistor. Appl Phys Lett.

[13] Sun Y, Xie D, Zhang X, Xu J, Li X, Dai R, Li P, Gao X, Zhu H (2017). Temperature dependent transport and hysteretic behaviors induced by interfacial states in MoS_2_ field-effect transistors. Nanotechnol.

[14] Wang XD, Wang P, Wang JL, Hu WD, Zhou XH, Guo N, Huang H, Sun S, Shen H, Lin T, Tang MH, Liao L, Jiang AQ, Sun JL, Meng XJ, Chen XS, Lu W, Chu JH (2015). Ultrasensitive and broadband MoS_2_ photodetector driven by ferroelectrics. Adv Mater.

[15] Lee YT, Hwang DK (2015). High-performance a MoS_2_ nanosheet-based nonvolatile memory transistor with a ferroelectric polymer and graphene source-drain electrode. J Korean Phys Soc.

[16] Lee HS, Min SW, Park MK, Lee YT, Jeon PJ, Kim JH, Ryu S, Im S (2012). MoS_2_ nanosheets for top-gate nonvolatile memory transistor channel. Small.

[17] Kobayashi T, Hori N, Nakajima T, Kawae T (2016). Electrical characteristics of MoS_2_ field-effect transistor with ferroelectric vinylidene fluoride-trifluoroethylene copolymer gate structure. Appl Phys Lett.

[18] Yap WC, Jiang H, Liu J, Xia Q, Zhu W (2017). Ferroelectric transistors with monolayer molybdenum disulfide and ultra-thin aluminum-doped hafnium oxide. Appl Phys Lett.

[19] Lipatov A, Sharma P, Gruverman A, Sinitskii A (2015). Optoelectrical molybdenum disulfide (MoS_2_)—ferroelectric memories. ACS Nano.

[20] Lyu X, Si M, Sun X, Capano MA, Wang H, Ye PD (2019) Ferroelectric and anti-ferroelectric hafnium zirconium oxide: scaling limit, switching speed and record high polarization density. VLSI Technology, Symposium on IEEE

[21] Yu Z, Wang H, Li W, Xu S, Song X, Wang S, Wang P, Zhou P, Shi Y, Chai Y, Wang X (2018) NC 2D MoS2 transistors with sub-60 mV/dec subthreshold swing over 6 orders, 250 μA/μm current density, and nearly-hysteresis-free. Electron Devices Meeting IEEE

[22] Mengwei S, Ye PD (2018) Steep slope 2D NC CMOS devices: MoS2 and WSe2. VLSI Technology, Symposium on IEEE

[23] Si M, Su CJ, Jiang C, Conrad NJ, Zhou H, Maize KD, Qiu G, Wu CT, Shakouri A, Alam MA, Ye PD (2012). Steep slope hysteresis-free NC MoS_2_ transistors. Nat Nanotechnol.

[24] Si M, Jiang C, Su CJ, Tang YT, Yang L, Chung W, Alam MA, Ye PD (2017) Sub-60 mV/dec ferroelectric HZO MoS2 NC field-effect transistor with internal metal gate: the role of parasitic capacitance. Electron Devices Meeting IEEE

[25] Mcguire FA, Lin YC, Price KM, Rayner GB, Khandelwal S, Salahuddin S (2017). Sustained sub-60 mV/decade switching via the NC effect in MoS_2_ transistors. Nano Lett.

[26] Nourbakhsh A, Zubair A, Joglekar S, Dresselhaus MS, Palacios T (2017). Subthreshold swing improvement in MoS_2_ transistors by the negative-capacitance effect in a ferroelectric Al-doped-HfO_2_/HfO_2_ gate dielectric stack. Nanoscale.

[27] Li H, Zhang Q, Yap CCR, Tay BK, Edwin THT, Olivier A, Baillargeat D (2012). From bulk to monolayer MoS_2_ evolution of Raman scattering. Adv Funct Mater.

[28] Moulder JF, Stickle WF, Sobol PE, Bomben KD (1995) Handbook of X-ray photoelectron spectroscopy: a reference book of standard spectra for identification and interpretation of XPS data. Perkin-Elmer Corporation, USA

[29] Zhou J, Han G, Li J, Liu Y, Peng Y, Zhang J (2018). Effects of the variation of V_GS_ sweep range on the performance of NCFETs. IEEE Electron Device Lett.

[30] Pahwa G, Dutta T, Agarwal A, Khandelwal S, Salahuddin S, Hu C (2016). Analysis and compact modeling of NC transistor with high on-current and negative output differential resistance-part ii: model validation. IEEE Trans Electron Devices.

[31] Krivokapic Z, Rana U, Galatage R, Razavieh A, Aziz A, Liu J, Shi J, Kim HJ, Sporer R, Serrao C, Busquet A, Polakowski P, Müller J, Kleemeier W, Jacob A, Brown D, Knorr A, Carter R, Banna S (2017) 14 nm ferroelectric FinFET technology with steep subthreshold slope for ultra low power applications. Electron Devices Meeting IEEE

[32] Zhou J, Wu J, Han G, Kanyang R, Peng Y, Li J, Wang H, Liu Y, Zhang J, Sun QQ, Zhang WD, Hao Y (2017) Frequency dependence of performance in Ge NC pFETs achieving sub-30 mV/decade swing and 110 mV hysteresis at MHz. Electron Devices Meeting IEEE

[33] Zhou J, Han G, Li Q, Peng Y, Lu X, Zhang C, Zhang J, Sun QQ, Zhang DW, Hao Y (2016) Ferroelectric HfZrOx Ge and GeSn pMOSFETs with sub-60 mV/decade subthreshold swing, negligible hysteresis, and improved Ids. Electron Devices Meeting IEEE

[34] Salahuddin S, Datta S (2008). Use of NC to provide voltage amplification for low power nanoscale devices. Nano Lett.

[35] Zhou J, Han G, Xu N, Li J, Peng Y, Liu Y (2019). Incomplete dipoles flipping produced near hysteresis-free NC transistors. IEEE Electron Device Lett.

[36] Zubko P, Wojdel JC, Hadjimichael M, Fernandezpena S, Sene A, Lukyanchuk I, Triscone JM, Iniguez J (2016). NC in multidomain ferroelectric superlattices. Nature.

[37] Kim YJ, Park MH, Lee YH, Kim HJ, Jeon W, Moon T, Kim KD, Jeong DS, Yamada H, Hwang CS (2016). Frustration of NC in Al_2_O_3_/BaTiO_3_ bilayer structure. Sci Rep.

[38] Wong JC, Salahuddin S (2018). NC transistors. Proc IEEE.

[39] Khan AI (2018). On the microscopic origin of NC in ferroelectric materials: a toy model.

[40] Zhou J, Han G, Xu N, Li J, Peng Y, Liu Y, Zhang J, Sun Q, Zhang DW, Hao Y (2019). Experimental validation of depolarization field produced voltage gains in NC field-effect transistors. IEEE Trans Electron Devices.

[41] Rusu A, Salvatore G A, Jiménez D, Ionescu AM (2011) Metalferroelectric-meta-oxide-semiconductor field effect transistor with sub-60 mV/decade subthreshold swing and internal voltage amplification. Electron Devices Meeting IEEE

[42] Zhou J, Han G, Xu N, Li J, Peng Y, Liu Y, Zhang J, Sun Q, Zhang DW, Hao Y (2017). Comparative study of NC Ge pFETs with HfZrO_x_ partially and fully covering gate region. IEEE Trans Electron Devices.

[43] Agarwal H, Kushwaha P, Lin Y, Kao M, Liao Y, Dasgupta A, Salahuddin S, Hu C (2019). Proposal for capacitance matching in NC field-effect transistors. IEEE Electron Device Lett.

[44] Li J, Zhou J, Han G, Liu Y, Peng Y, Zhang J, Sun Q, Zhang DW, Hao Y (2018) NC Ge pFETs for performance improvement: impact of thickness of HfZrOx. IEEE Trans Electron Devices PP(99):1-6

[45] Gajal L, Kumar N, Amin SI, Anand S (2020). Design and performance enhancement of doping less feld effect transistor with the help of NC technique. Appl Phys A Mater Sci Process.

[46] Zhou H, Kwon D, Sachid A B, Liao Y, Salahuddin S (2018) NC, n-Channel, Si FinFETs: bi-directional sub-60 mV/dec, negative DIBL, negative differential resistance and improved short channel effect. VLSI Technology, Symposium on IEEE

[47] Kwon D, Chatterjee K, Tan AJ, Yadav AK, Zhou H, Sachid AB (2018). Improved subthreshold swing and short channel effect in FDSOI n-channel negative capacitance field effect transistors. IEEE Electron Device Lett.

[48] Late DJ, Liu B, Matte HSSR, David VP, Rao CNR (2012). Hysteresis in single-layer MoS_2_ field effect transistors. ACS Nano.

[49] Schenk T, Schroeder U, Pesic M, Popovici M, Pershin YV, Mikolajick T (2014). Electric field cycling behavior of ferroelectric hafnium oxide. ACS Appl Mater Interfaces.

[50] Menou N, Muller C, Baturin IS, Shur VY, Pershin YV, Hodeau JL (2005). Polarization fatigue in PbZr0.45Ti0.55O3-based capacitors studied from high resolution synchrotron X-ray diffraction. J Appl Phys.

[51] Gong N, Ma TP (2016). Why is FE–HfO_2_ more suitable than PZT or SBT for scaled nonvolatile 1-T memory cell? A retention perspective. IEEE Electron Device Lett.

[52] Horiuchi T, Takahashi M, Li QH, Wang S, Sakai S (2010). Lowered operation voltage in Pt/SBi_2_Ta_2_O_9_/HfO_2_/Si ferroelectric-gate field-effect transistors by oxynitriding Si. Semicond Sci Technol.

[53] Yurchuk E, Müller J, Müller S, Paul J, Mikolajick T (2016). Charge-trapping phenomena in HfO_2_-based FeFET-type nonvolatile memories. IEEE Trans Electron Devices.

[54] Zhou J, Han G, Peng Y, Liu Y, Zhang J, Sun QQ (2017). Ferroelectric NC GeSn pFETs with sub-20 mV/decade subthreshold swing. IEEE Electron Device Lett.

[55] Jo J, Shin C (2016). NC field effect transistor with hysteresis-free sub-60-mV/decade switching. IEEE Electron Device Lett.

[56] Zhou J, Peng Y, Han G, Li Q, Hao Y (2017). Hysteresis reduction in NC Ge pFETs enabled by modulating ferroelectric properties in HfZrOx. IEEE J Electron Device Soc.

[57] Chung W, Si M, Ye PD (2017) Hysteresis-free NC germanium CMOS FinFETs with bi-directional sub-60 mV/dec. Electron Devices Meeting IEEE

[58] Lee SY, Chen HW, Shen CH, Kuo PY, Chung CC, Huang YE, Chen HY, Chao TS (2020). Effect of seed layer on gate-all-around poly-Si nanowire negative-capacitance FETs with MFMIS and MFIS structures: planar capacitors to 3-D FETs. IEEE Trans Electron Devices.

[59] Agarwal H, Kushwaha P, Duarte JP, Lin YK, Hu C (2018). Engineering negative differential resistance in NCFETs for analog applications. IEEE Trans Electron Devices.

[60] Li Y, Kang Y, Gong X (2017). Evaluation of NC ferroelectric MOSFET for analog circuit applications. IEEE Trans. Electron Devices.

[61] Gupta S, Steiner M, Aziz A, Narayanan V, Datta S, Gupta SK (2017). Device-circuit analysis of ferroelectric FETs for low-power logic. IEEE Trans Electron Devices.

