# Supplementary material for: Low Voltage Operating 2D MoS2 Ferroelectric Memory Transistor with Hf1-xZrxO2 Gate Structure
Source: Nanoscale Res Lett. 2020 Aug 2;15:157. doi: 10.1186/s11671-020-03384-z (PMC7396413; doi:10.1186/s11671-020-03384-z)
Supplement: Supplementary file 1 — Additional file 1: Supplementary Information. Characterization of ferroelectric HZO substrate and more transfer curves of the HZO/MoS2 FeFET(PDF). Fig. S1 a Optical image of the MoS2/HZO FeFET. bPRphase and cPRampl of the HZO capacitor. XPS analysis of the 2 nm Al2O3/6 nm HZO/p+ Si shows pristine d Hf and e Zr peaks. f XPS depth profile of the Al2O3/HZO/p+ Si tri-layer structure. A top-view optic image of the HZO/MoS2 FeFET is shown in Fig. S1a. As shown in Fig. S1b and c, PRphase and PRampl of the HZO capacitor suggest ferroelectric behavior after 450 °C rapid thermal annealing (RTA) measured at 1kHz. As shown in Fig. S1d and e, the elemental and bond composition of HZO were examined by the X-ray photoelectron (XPS) measurements. Peaks are found to be 19.05 eV, 17.6 eV, 185.5 eV, and 183.2 eV, which correspond to the Hf 4f 5/2, Hf 4f 7/2, Zr 3d 3/2 and Zr 3d 5/2, respectively [27]. The atomic concentration along the depth profile in Fig. S1f further confirms the distribution of the Al2O3/HZO/p+ Si tri-layer structure. All the above confirm that the HZO film grown via our ALD system is highly crystalline. Fig. S2. Transfer curves of the HZO/MoS2 FeFET at increasing gate voltage (VGS) ranges with the linear y-axis. For a start, the transfer curves of the HZO/MoS2 FeFET under different back gate voltage sweep ranges (VGS,range) and different drain voltages (VDS) have been characterized in Fig. S2. It is demonstrated that, the counterclockwise hysteresis windows have been obtained at various gate voltage range (VGS,range) from (-5, 5V) to (-2, 2V). Simply, the mechanism underlying the hysteretic behaviors shown in the transfer curves during the bi-direction sweeping of VGS is threshold voltage shift, which can be modified by the effects of trapping/de-trapping [52] and polarization switching [53]. If the applied voltage is not high enough to switch the polarization in HZO film, charge trapping/de-trapping mechanism dominates and will cause clockwise hysteresis. The ene [file 11671_2020_3384_MOESM1_ESM.docx]

**Supporting Information for:**

**Low Voltage Operating 2D MoS_2_ Ferroelectric Memory Transistor with Hf_1-x_Zr_x_O_2_ Gate Structure**

Siqing Zhang^1^, Yan Liu^1,^*, Jiuren Zhou^1^, Meng Ma^1^, Anyuan Gao^2^, Binjie Zheng^2^, Lingfei Li^2^, Xin Su^2^, Genquan Han^1^, Jincheng Zhang^1^, Yi Shi^2^, Xiaomu Wang^2,^*, and Yue Hao^1^

^1^Wide Bandgap Semiconductor Technology Disciplines State Key Laboratory, School of Microelectronics, Xidian University, Xi’an 710071, China

^2^School of Electronic Science and Engineering, Nanjing University, Nanjing, Jiangsu, 210023, China

* Corresponding authors.

E-mail addresses: xdliuyan@xidian.edu.cn (Y Liu), [xiaomu.wang@nju.edu.cn](mailto:xiaomu.wang@nju.edu.cn) (X Wang).


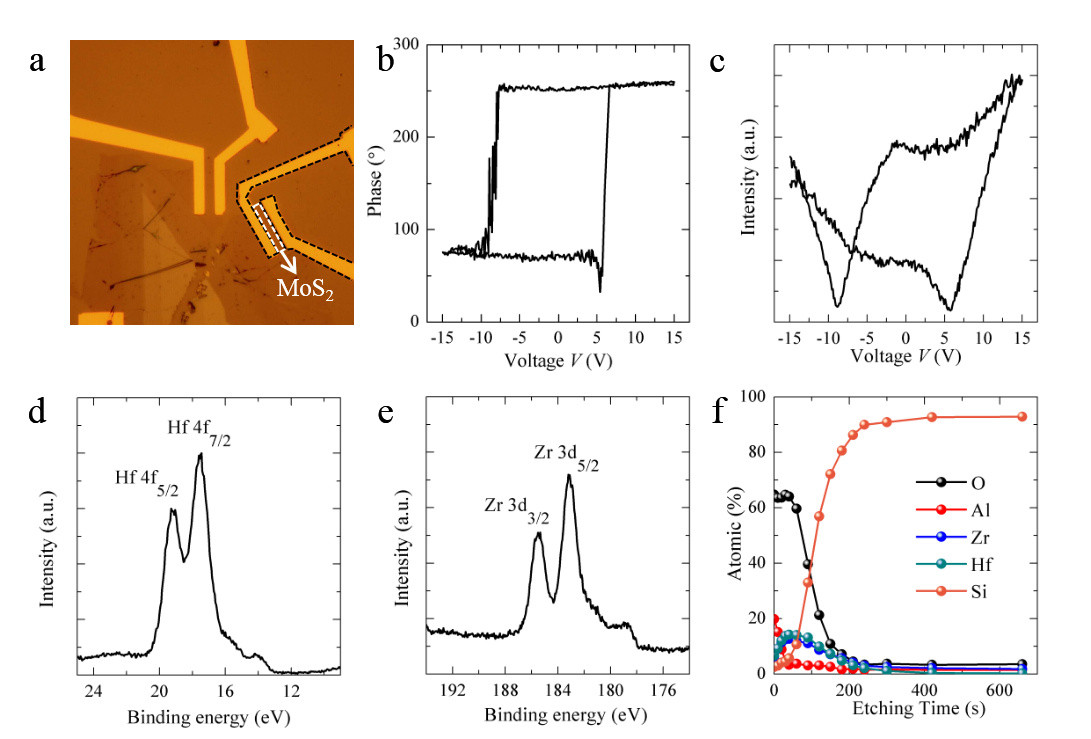


**Fig. S1** **a** Optical image of the MoS_2_/HZO FeFET. **b** *PRphase* and **c** *PRampl* of the HZO capacitor. XPS analysis of the 2 nm Al_2_O_3_/6 nm HZO/p^+^ Si shows pristine **d** Hf and **e** Zr peaks. **f** XPS depth profile of the Al_2_O_3_/HZO/p^+^ Si tri-layer structure.

A top-view optic image of the HZO/MoS_2_ FeFET is shown in Fig. S1a. As shown in Fig. S1b and c, *PRphase* and *PRampl* of the HZO capacitor suggest ferroelectric behavior after 450 ℃ rapid thermal annealing (RTA) measured at 1kHz. As shown in Fig. S1d and e, the elemental and bond composition of HZO were examined by the X-ray photoelectron (XPS) measurements. Peaks are found to be 19.05 eV, 17.6 eV, 185.5 eV, and 183.2 eV, which correspond to the Hf 4f _5/2_, Hf 4f _7/2_, Zr 3d _3/2_ and Zr 3d _5/2_, respectively [27]. The atomic concentration along the depth profile in Fig. S1f further confirms the distribution of the Al_2_O_3_/HZO/p^+^ Si tri-layer structure. All the above confirm that the HZO film grown via our ALD system is highly crystalline.


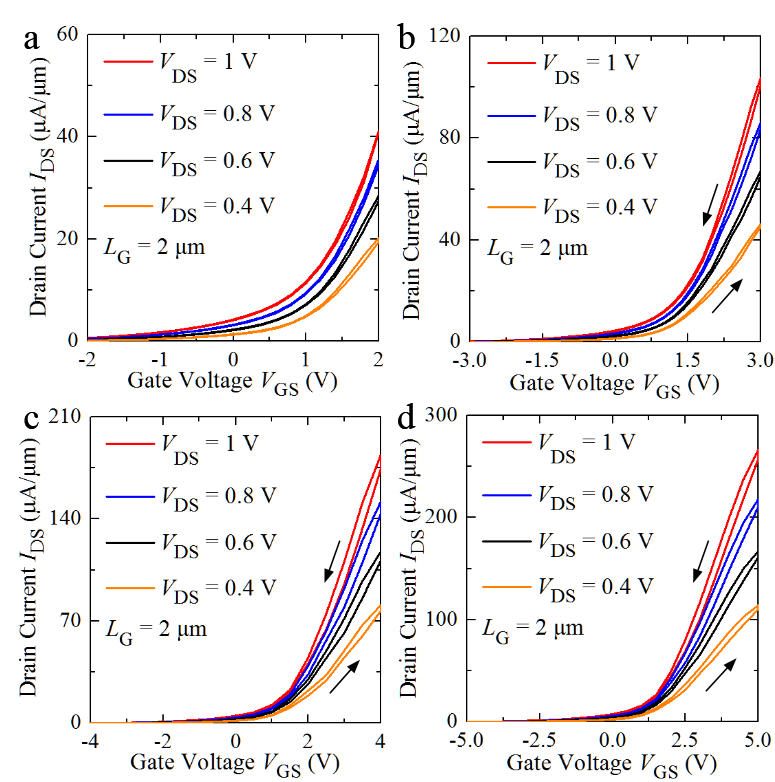


**Fig. S2** Transfer curves of the HZO/MoS_2_ FeFET at increasing gate voltage (*V*_GS_) ranges with the linear y-axis.

For a start, the transfer curves of the HZO/MoS_2_ FeFET under different back gate voltage sweep ranges (*V*_GS,range_) and different drain voltages (*V*_DS_) have been characterized in Fig. S2. It is demonstrated that, the counterclockwise hysteresis windows have been obtained at various gate voltage range (*V*_GS,range_) from (-5, 5V) to (-2, 2V). Simply, the mechanism underlying the hysteretic behaviors shown in the transfer curves during the bi-direction sweeping of *V*_GS_ is threshold voltage shift, which can be modified by the effects of trapping/de-trapping [29] and polarization switching [30]. If the applied voltage is not high enough to switch the polarization in HZO film, charge trapping/de-trapping mechanism dominates and will cause clockwise hysteresis. The energy band at the interface between the MoS_2_ channel and ferroelectric back gate tends to bend downward after the positive back gate voltage. The more traps located below the Fermi-level; the more electrons are captured close to the interface. This will increase the threshold voltage after the positive gate pulse. The energy band at the interface between the MoS_2_ channel and ferroelectric back gate tends to bend upward after the negative back gate voltage. The more traps locate above the Fermi-level; the more electrons are released close to the interface. This will decrease the threshold voltage after the negative gate pulse [29]. If the applied voltage exceeds the coercive voltage in the HZO film, ferroelectric polarization mechanism dominates and will cause anti-clockwise hysteresis window [31-34]. Thus, it is easily concluded that the electrical performance of the device shown in Fig. S2 is dominated by ferroelectric switching. When the back-gate sweeps are in small ranges of 2V in Fig. S2a, we observed the nearly hysteresis-free switching. The hysteresis loops in Fig. S2b are counterclockwise for the back-gate sweep range of 6 V (from -3 V to 3 V). The minimum voltage under the drain is *V*_GS_ – *V*_DS_ = 2 V at *V*_DS_ = 1 V, which should be larger than the coercive voltage *V*_c_ to switch the ferroelectric at the drain side. The estimated coercive voltage is consistent with *V*_c_ of 1.9 V when the maximum sweeping voltage is 3 V in Fig. 2a. When the applied voltage in HZO film exceeds +*V*_c_, the ferroelectric polarization points into the MoS_2_ channel. Therefore, the electron charges in the MoS_2_ channel accumulate and the threshold voltage decreases. When the applied voltage in HZO film exceeds –*V*_c_, the ferroelectric polarization points away from the MoS_2_ channel. Therefore, the electron charges in the MoS_2_ channel deplete and the threshold voltage increases. Nonetheless, we observed that the wider back-gate voltage range leads to larger counterclockwise hysteresis loops in Fig. S2c and d. Due to the increment of *V*_c_ in Fig. 2a with increasing applied voltage, the ferroelectric polarization switching in the HZO film can be enhanced with a larger shift in threshold voltage.


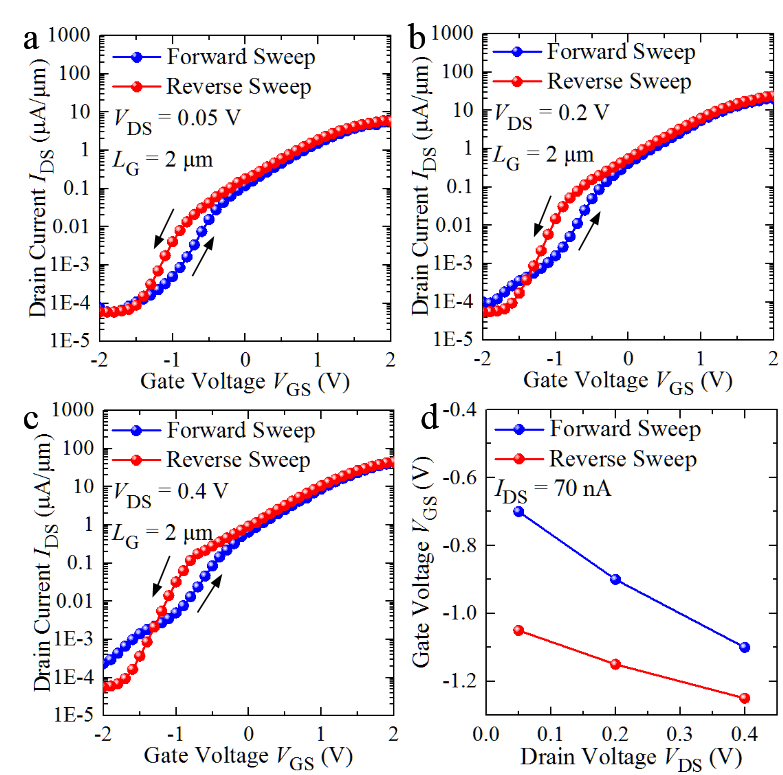


**Fig. S3** Transfer curves of the HZO/MoS_2_ FeFET on logarithmic scales with **a** *V*_DS_ = 0.05 V, **b** *V*_DS_ = 0.2 V, **c** *V*_DS_ = 0.4 V. **d** Extracted back gate voltage *V*_GS_ when drain current (*I*_DS_) equals to 70 nA with different *V*_DS_.

Notably, besides the impact of *V*_GS,range_, it is found that *V*_DS_ can definitely adjust the memory window as well, which requires a further investigation. The *I*_DS_-*V*_GS_ curves on logarithmic scales under different *V*_DS_ are characterized in Fig. S3. It is exhibited that, at a fixed *V*_GS,range_ = (-2, 2 V), the values of *V*_GS_ extracted at *I*_DS_ = 70 nA for the bi-directional sweeping of *V*_GS_ all shift towards the negative direction and the variation in forward sweeping of *V*_GS_ is much more obvious over that of reverse sweeping, indicating the significant phenomena of negative drain induced barrier lowering (DIBL) [36-40]. Generally, DIBL is a conventional short channel effect. With a short enough channel length, the increased *V*_DS_ can easily pull down the barrier between source/drain and enable a negative shift of threshold voltage, which is the so called effect of DIBL. However, for a ferroelectric FeFET, an increased *V*_DS_ is capable of producing a reduction of channel surface potential via the coupling between gate and drain induced by the parasitic capacitance between gate and drain (CGD), which means a positive shift of threshold voltage and can be called as negative DIBL.
